# Absorbance or organization into ankylosis: a microarray analysis of haemarthrosis in a sheep model of temporomandibular joint trauma

**DOI:** 10.1186/s12903-021-02033-w

**Published:** 2021-12-28

**Authors:** Mai-Ning Jiao, Tong-Mei Zhang, Kun Yang, Zhao-Yuan Xu, Guan-Meng Zhang, Yuan-Yuan Tian, Hao Liu, Ying-Bin Yan

**Affiliations:** 1 Tianjin Medical University, 22 Qi-xiang-tai Road, Heping District, Tianjin, 300070 People’s Republic of China; 2 Department of Oromaxillofacial-Head and Neck Surgery, Tianjin Stomatological Hospital, 75 Dagu Road, Heping District, Tianjin, 300041 People’s Republic of China; 3 Tianjin Key Laboratory of Oral and Maxillofacial Function Reconstruction, 75 Dagu Road, Heping District, Tianjin, 300041 People’s Republic of China; 4 Department of Oral and Maxillofacial Surgery, China Three Gorges University Affiliated Renhe Hospital, 410 Yiling Ave, Hubei, 443001 People’s Republic of China

**Keywords:** Temporomandibular ankylosis, Trauma, Hematoma, Microarray analysis, Sheep

## Abstract

**Background:**

Traumatic haemarthrosis was hypothesized to be the etiology of temporomandibular (TMJ) ankylosis. Here, taking haematoma absorbance as a control, we aimed to reveal the molecular mechanisms involved in haematoma organizing into ankylosis using transcriptome microarray profiles.

**Material/methods:**

Disk removal was performed to building haematoma absorbance (HA) in one side of TMJ, while removal of disk and articular fibrous layers was performed to induced TMJ ankylosis through haematoma organization (HO) in the contralateral side in a sheep model. Haematoma tissues harvested at days 1, 4 and 7 postoperatively were examined by histology, and analyzed by Affymetrix OviGene-1_0-ST microarrays. The DAVID were recruited to perform the Gene Ontology and Kyoto Encyclopedia of Genes and Genomes pathway analysis for the different expression genes (DEGs). The DEGs were also typed into protein–protein interaction (PPI) networks to get the interaction data. Six significant genes screened from PPI analysis, were confirmed by real-time PCR.

**Results:**

We found 268, 223 and 17 DEGs at least twofold at days 1, 4 and 7, respectively. At day 1, genes promoting collagen ossification (POSTN, BGN, LUM, SPARC), cell proliferation (TGF-β), and osteogenic differentiation of mesenchymal stem cells (BMP-2) were up-regulated in the HO side. At day 4, several genes involved in angiogenesis (KDR, FIT1, TEK) shower higher expression in the HO side. While HA was characterized by a continuous immune and inflammatory reaction.

**Conclusions:**

Our results provide a comprehensive understanding of the role of haematoma in the onset and progress of TMJ ankylosis. The study will contribute to explaining why few injured TMJs ankylose and most do not from the molecular level.

**Supplementary Information:**

The online version contains supplementary material available at 10.1186/s12903-021-02033-w.

## Introduction

Temporomandibular joint (TMJ) ankylosis refers to bone or fibrous adhesion of the anatomic joint components with the ensuing progressive limitation of mouth opening [1]. Trauma is the most important cause of the disease, account for 31–98% cases [2]. Although for most patients with ankylosis, the primary trauma was severe enough to produce a condylar fracture, traumatic haemarthrosis, rather than the condylar fracture itself, was hypothesized to be the etiology of TMJ ankylosis [3]. The reasons were as follows: 1. cases with traumatic TMJ ankylosis but without condylar fracture have been reported [3]; 2. some patients developed into TMJ ankylosis after arthroscopy [4]; and 3. only a very few fractured TMJs ankylosed (0.4–2%) and most did not [3].

However, the outcome of experimental haemarthrosis by injection of blood into the articular space was absorbance, rather than TMJ ankylosis [3, 5]. Similarly, the arthroscopic examination of 20 patients with acute TMJ trauma demonstrated that haemarthrosis rapidly spontaneously resolved by 5–7 days [6]. These results indicated that haemarthrosis alone was not sufficient for the formation of ankylosis. Recently, we found that experimental haemarthrosis induced by discectomy resolved and no TMJ ankylosis occurred 3 months postoperatively in a sheep model [7, 8]. However, if the discectomy combined with removal of the fibrous layers covering on the condyle and the glenoid fossa, haemarthrosis organized and developed into ankylosis inevitably [7].

Our findings suggested a couple of things. Firstly, since removal of the fibrous layers will lead to exposure of the underneath proliferative zone cells or chondrocytes on the articular surfaces according to the TMJ histology and our previous animal study [9], the fate of experimental haemarthrosis, whether absorbance or organization into ankylosis, depended on the tissue it contacted in the animal model. Secondly, although several previous studies had explored the molecular pathophysiology of TMJ ankylosis in animal models [10, 11], these studies, in fact, tried to answer how the haemarthrosis developed into ankylosis in a specific traumatic microenvironment, rather than elucidate the molecular basis why some of haemarthrosis organizated into ankylosis, while most of them resolved. Taking haematoma absorbance as a control to explore the molecular pathogenesis of TMJ ankylosis, would contribute to answering the question mentioned above. Thirdly, since most of haemarthrosis after TMJ trauma rapidly resolved within 1 week for human beings [6], taking haematoma absorbance as a control would also contribute to uncovering the early stage of molecular pathophysiology of TMJ ankylosis, which is largely unknown.

Therefore the experimental haemarthrosis with or without removal of articular fibrous layers is an established and suitable model for gene expression profiling studies regarding the onset of TMJ ankylosis. Our group has previously used a condylar fracture model to analyze the differential gene expression profiles between traumatic TMJ fibrous and bony ankylosis [12]. Recently, Zhang et al. [13] also explored the role of lateral pterygoid muscle in the traumatic TMJ ankylosis by gene chip. However, up to now there have been no published reports of genome-wide transcriptional analysis of TMJ ankylosis that takes place in an experimental haemarthrosis models without condylar fracture. Our aim in this study is to compare the differential gene transcription profiles between haematoma absorbance (HA) and haematoma organization (HO) into ankylosis in a sheep model of TMJ trauma. These findings will provide useful information for elucidating the molecular mechanism regarding the onset of TMJ ankylosis.

## Materials and methods

### Animal model and tissue processing

Three-month-old male small-tailed Han sheep with body weight ranging from 25 to 27 kg were used in this study under a research protocol approved by the Ethics Committee of Tianjin Stomatological Hospital (Tjskq2013001). The animals were kept in an animal-care facility with strict compliance to care and usage protocols that were previously described [14, 15]. The animal model was established according to the protocol outlined in our previous publication [7]. The method of anesthesia, analgesia and euthanasia used for sheep was the same as described previously [12]. All animals received bilateral TMJ surgery. Disk removal was performed on one side, which has been shown to present as haematoma absorbance (HA) and the ensuing TMJ osteoarthritis, and removal of disk and articular fibrous layers was performed on the contralateral side, which has been shown to support hematoma organization (HO) and produce TMJ fibro-osseous ankylosis [7].

Since the time for absorbance of haemarthrosis in the animal model was unclear, 15 sheep were sacrificed at days 1, 4, 7, 9 and 11 postoperatively with 3 animals being killed per time point to explore the early stage of pathological process in experiment 1. After the sheep were sacrificed with a 120 mg/kg of pentobar-bitone sodium given through intravenous injection, the bilateral TMJs were opened carefully. The haematoma or newly formed tissue within the joint space was bluntly dissection from the surrounding soft tissue by periosteum separators. It is important to distinguish the haematoma located at the intracapsular or extracapsular site. Only the intracapsular haematoma or newly formed tissue was harvested for further analysis. The tissue was fixed in 10% natural buffered formalin for 72 h, then dehydrated and embedded in paraffin. Five-μm-thick slices were cut with a microtome, and the slides were examined after staining with hematoxylin–eosin (H-E).

According to the results of experiment 1, another 9 sheep were added and sacrificed at days 1, 4 and 7 after surgery with 3 animals being killed per time point in experiment 2. The site of tissue harvest was the same as experiment 1. The tissue was rapidly frozen in liquid nitrogen and stored at −80 ℃ for ribonucleic acid (RNA) extraction and the subsequent microarray analysis.

### RNA preparation and microarray data acquisition

RNA preparation and microarray detection were performed by CNKingBio Corporation (Beijing, China). In brief, we extracted total RNA from the hematoma using Trizol reagent (Invitrogen Life Technologies, Carlsbad, USA) and using the RNEasy small kit (Qiagen, Valencia, USA) purified total RNA. Biotinylated complementary deoxyribonucleic acid (cDNA) was then prepared from 150 ng total RNA using an Ambion®WT expression kit according to the Affymetrix standard protocol. After tagging, fragments of cDNA were hybridized at 45 °C for 16 h on Affymetrix® Ovigene-1_0-ST Array (Affymetrix), a microarray containing over 22,047 known transcripts and probe sets of expressed sequence tags. The gene chip was cleaned and stained in the Affymetrix Fluidics Station 450. All arrays were scanned by the Affymetrix® Genchip Command Console (AGCC), which was installed in the Genchip ®Scanner 3000 7G.

### Microarray expression data analysis

#### Identification of different expression genes (DEGs)

The raw expression data were first background corrected and quantile normalized by the robust multichip analysis (RMA) algorithm using Affymetrix default analysis settings. Values presented were log_2_ RMA signal intensity. The fold-change (FC) was calculated with HA as control. DEGs were identified using limma package [16] in R/Bioconductor (https://bioconductor.org/packages/release/bioc/html/limma.html) for comparison of the HA and HO with the cutoff value of FC > 2 and *p* < 0.05. We used volcano plot and heatmap to show the distribution of DEGs intuitively. The volcano plot and heatmap of the DEGs were drawn via ggplot2 and heatmap packages in R software [17].

#### Function enrichment analysis

The gene ontology (GO) term enrichment (http://www.geneontology.org/) was utilized to group the identified DEGs into defined categories of cellular component (CC), molecular function (MF) and biological process (BP) and determine which genes were significant [18]. Kyoto encyclopedia of genes and genomes (KEGG) PATHWAY (http://www.genome.jp/kegg/pathway.html) was selected as the reference database for manually drawn pathway mapping [19]. The database for annotation, visualization and integration discovery (DAVID, version 6.8, http://david.abcc.Ncifcrf.gov/) [20] was recruited to perform the GO and KEGG pathway enrichment analysis and ascertained the functions and pathways that might be disturbed by the identified DEGs, with the selected criterion of a *p* < 0.05.

#### Protein–protein interaction network construction and module analysis

To evaluate the interactive relationships among DEGs, the protein–protein interaction pairs were executed using the STRING (Search Tool for the Retrieval of Interacting Genes, version 11.0; https://string-db.org/) [18], which is an online database to assess and integrate physical and functional protein–protein associations with species limited to “Ovis aries” and an interaction score confidence > 0.4 [21]. In PPI network, “node” represents a gene or protein, and “line” represents an interaction between the two nodes. The degree of each node (number of interactions with other proteins) is equal to the number of nodes that interacted with this node.

Significant modules in the PPI network were identified by molecular complex detection (MCODE) [22], a plug-in of Cytoscape software that clusters a network based on topology to recognize closely connected regions. The MCODE algorithm sorts and identifies each identified module. The higher the score is, the stronger the genes’ association in this module. The parameters of DEGs clustering and scoring were set as follows: degree cutoff = 2, node score cutoff = 0.2, K-Core = 2, and max depth = 100.

### Reverse transcription and real-time polymerase chain reaction (PCR)

Six interesting DEGs were selected from the microarray results to further confirm the reliability of the array data. Total RNA was isolated using Trizol Reagent (Invitrogen Life Technologies, Carlsbad, USA) according to the manufacturer’s instructions. Reverse transcription was performed with a cDNA synthesis kit (Promega, USA) in a 20 μl reaction system containing 2 μg total RNA [10].

Primers for each target gene were designed using the software of Primer Premier Version 5.0 and synthesized by Sangon Biotech (Shanghai) Co., Ltd, which were listed in Table 1. Quantitative real-time PCR was performed with FastStart Universal SYBR Green Master (Roche, ref. 04913850001) using LightCycler 480 II Instrument (Roche, Switzerland). The reaction system and PCR cycle parameters were the same as previously described [14]. The housekeeping glyceraldehyde-3-phosphate dehydrogenase (GAPDH) gene was used for normalization of target genes expression. Relative mRNA expressions of the target genes between the HA and HO were calculated using 2^−ΔΔct^ method as previously described [14].

**Table 1:** Real-time PCR primer sequence

Gene	Gene bank number	Primer sequences (5′–3′)	Product size (bp)
GAPDH	AF030943.1	GCAAGTTCCACGGCACAG	249
		GGTTCACGCCCATCACAA	
BGN	XM_015104664.2	GGAAGGGTCTCTTGGGGTGC	170
		TCAAGGGGCATGTGATGGGG	
KDR	XM_012179355.2	ATGGGAACCGAAACCTA	126
		CCTGGGCACCTTCTACT	
POSTN	XM_004012108.4	CCCCATAACTGTCTACAAGCCA	199
		TCTCACAGGTGTGTCTTCTTGC	
CXCL12	XM_012105583.1	CCTTGCCGATTCTTTGAG	189
		AGTGGGACTGGGTTTGTTT	
SPARC	XM_012177565.3	CTGGACTACATCGGGCCTTG	156
		CAGCTTCTGCTTCTCGGTCA	
TEK	XM_012127828.2	TCTGTGAAGGGCGAGTT	196
		GGCACCGAGTGGATGAA	

### Statistical analysis

The values of the HA and HO were compared at each time point. Independent *t* test was conducted using SPSS 17.0 (SPSS Inc., Chicago, IL) to determine whether statistical significance existed at a *p* value < 0.05.

## Results

### Gross appearance of haematoma

**Fig. 1:**
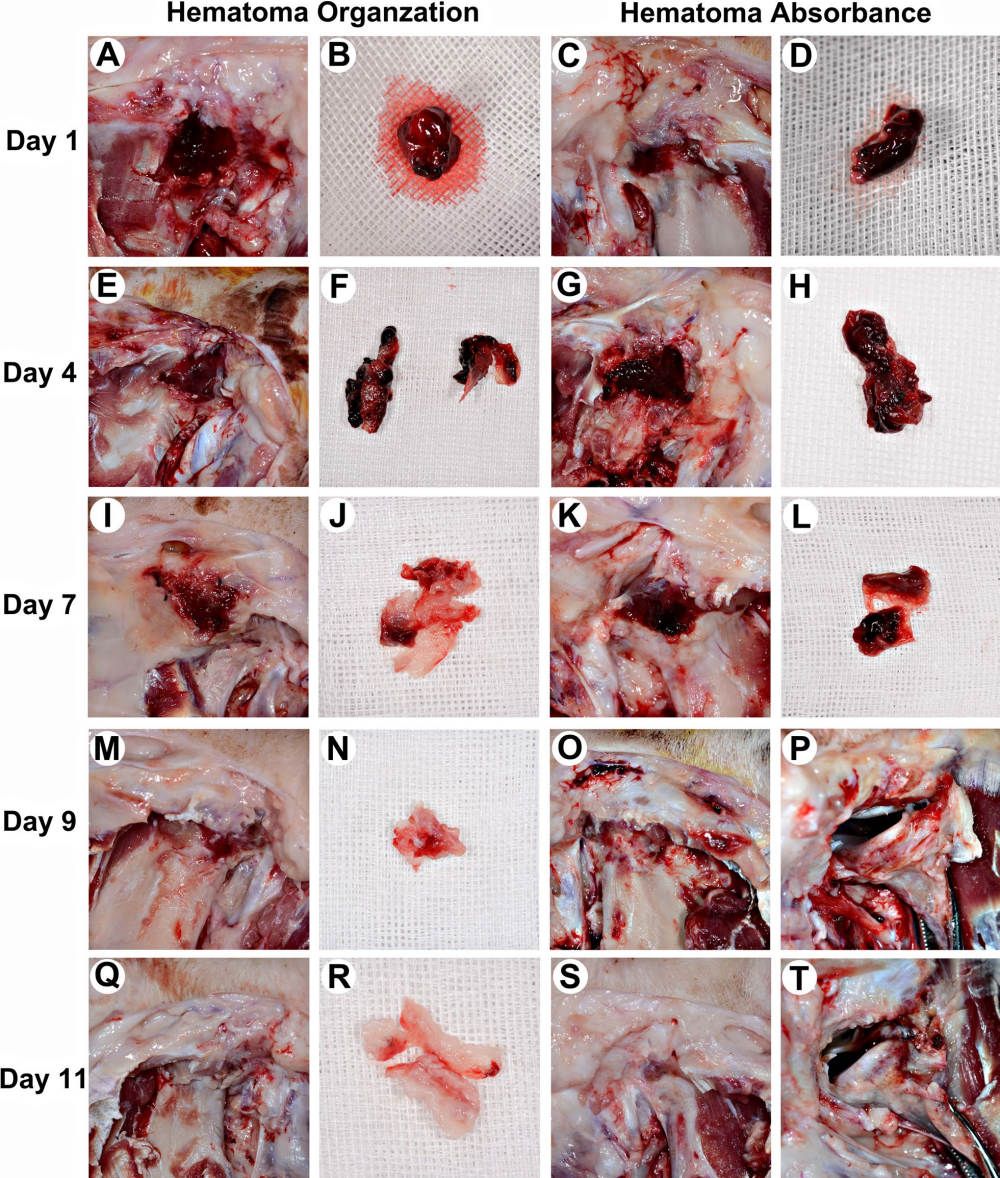
The gross appearance of the temporomandibular joint and hematoma at each postoperative time. **A**–**L** At day 1, 4 and 7 postoperatively, the appearance of temporomandibular joint area and hematoma samples of HO and HA side. At these time points, bilateral intraarticular hematomas were all visible to the naked eye. **M**, **N**, **Q**, **R** The performance of temporomandibular joint area and hematoma samples at day 9 and 11 of HO side. The hematoma has been organized on this side, and the new generated tissue filled in the joint space. **O**, **P**, **S**, **T** The appearance of the temporomandibular joint area and how it looks when it's opened at day 9 and 11 of HA side, we can only see the empty temporal mandibular joint cavity without new generated tissue. *HO* hematoma origination, *HA* hematoma absorbance

At days 1 and 4, large haematoma could be found by naked-eye in and around the joint cavities both in HA and HO sides (Fig. 1A–H). At day 7, the haematoma could also be observed in and around the joint cavities in the HO side (Fig. 1I, J). However the main part of haematoma in the HA side located at the extracapsule (Fig. 1K, L), indicating that it was almost absorbed in the joint space. At days 9 and 11, the haematoma in the HO side organized with the color changing from dark red to pink, and the new generated tissue filled in the lateral joint space (Fig. 1M, N, Q, R). While in the HA side, only a very small part of residual haematoma could be seen around the joint capsule but completely disappeared in the joint space without new generated tissue (Fig. 1O, P, S, T).

### Histological examination

**Fig. 2:**
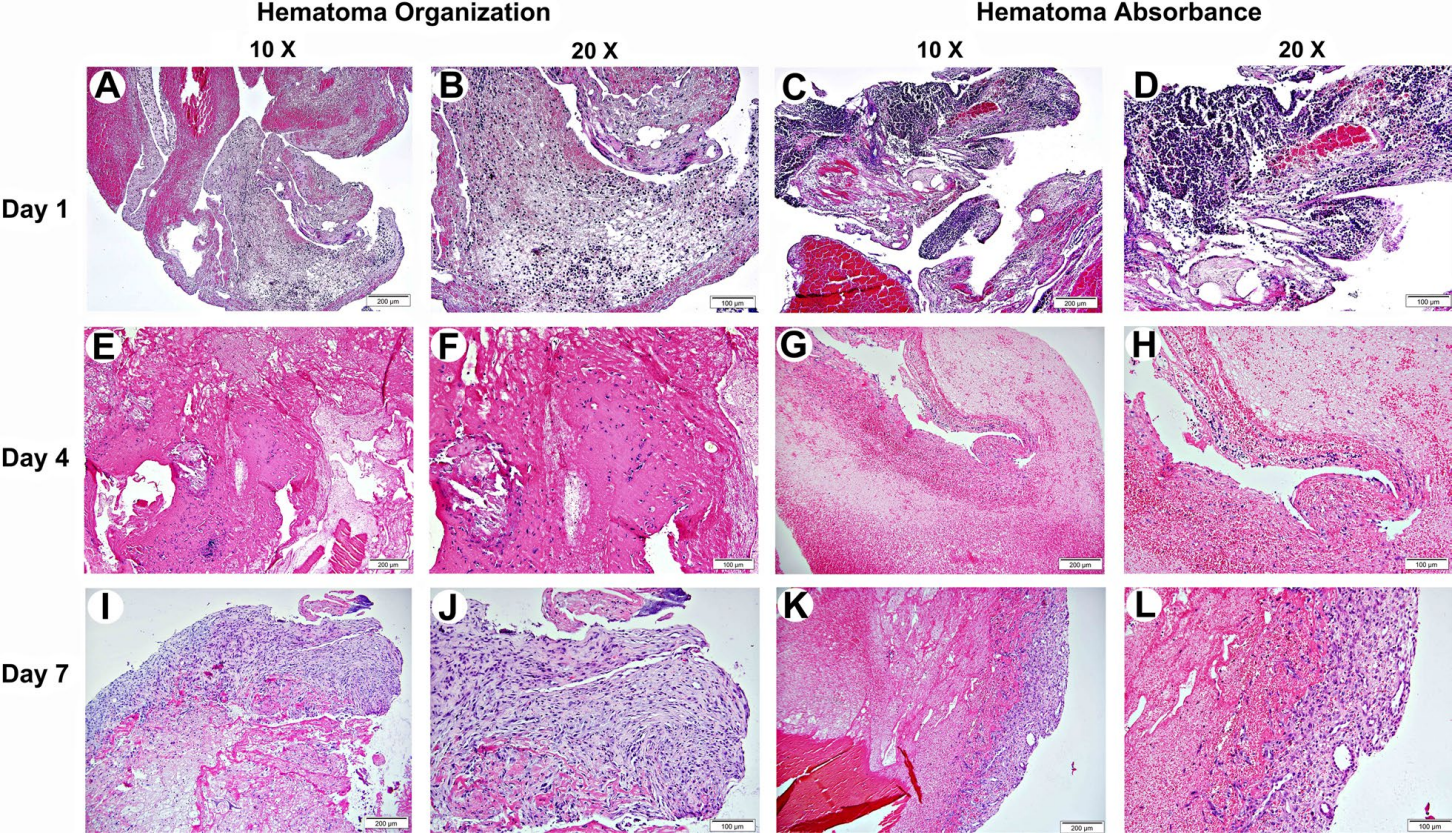
Photomicrographs of histological sections depicting the temporomandibular joint hematoma at day 1, 4, 7 of HO side and HA side. **A**–**D** Blood clots filled the bilateral articular space at day 1 after surgery. **E**–**H** At day 4 after surgery, a small amount of granulation began to be found on the HO side but not in the HA side. **I**–**L** At day 7 after surgery, granulation is present on both sides, but the HA side has a smaller range and size of granulation. Hematoxylin and eosin stain. **A**, **C**, **E**, **G**, **I** and **K** magnification ×10; scale bar 200 μm. **B**, **D**, **F**, **H**, **J** and **L** magnification ×20; scale bar 100 μm. *HO* hematoma origination, *HA* hematoma absorbance

Due to the haematoma in the joint space was completely absorbed at days 9 and 11 in the HA side, we could only compare the histology between the HA and HO at days 1, 4, and 7, and the results were illustrated in Fig. 2.

The first day after operation, blood clots filled the bilateral articular space, producing a fibrin and platelet laden with substrate for a succession of cellular infiltrates. Inflammatory cells were ubiquitous in the haematoma tissue, most of which were lobulated neutrophils. The degree of inflammation in the HA side was more severe than that in the HO side (Fig. 2A–D).

At day 4 after surgery, the haematoma was partially organized in the HO side. Besides erythrocytes and network of fibrin, a small amount of granulation tissue was found with blood vessels being detected (Fig. 2E, F). As a contrast, no granulation tissue was observed in the HA side, instead of a loose network of fibrin containing massive erythrocytes and mononuclear inflammatory cells (Fig. 2G, H).

At day 7, the haematoma was highly organized by a fibrin scaffold and large amounts of granulation tissue, with angiogenesis and increased fibroblasts in the HO side (Fig. 2I, J). Surprisingly, granulation tissue was also found in the HA side although its range and size were much lower than that in the HO side (Fig. 2K, L). Combined with the gross appearance that the main part of haematoma in the HA side located at the extracapsule at day 7, we speculated that the function of the granulation tissue was to repair the injured articular capsule.

### DEGs between HO and HA samples

**Fig. 3:**
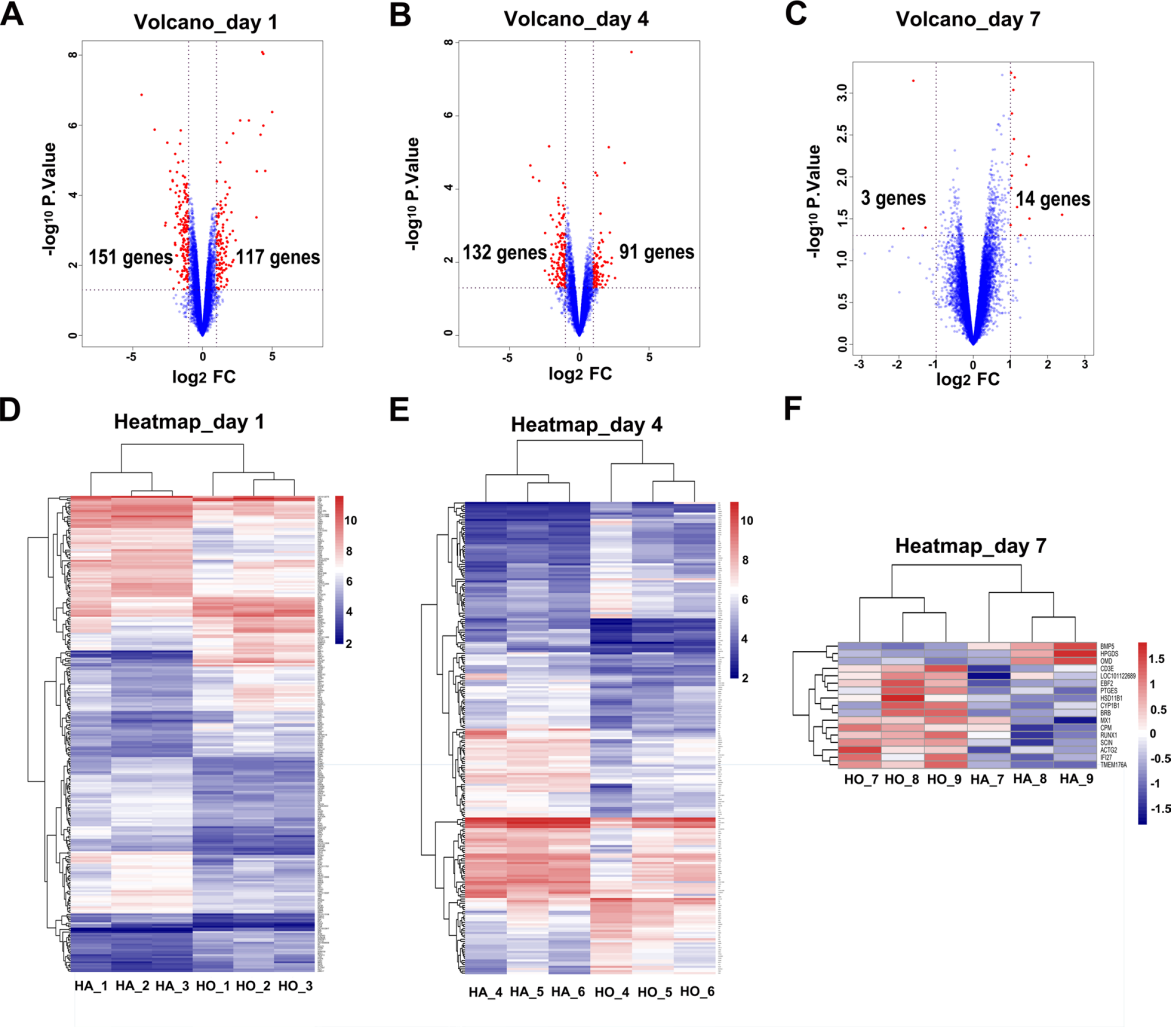
DEGs (fold change > 2, *p* < 0.05) in the microarray profiles. **A** Volcano plot of 3 paired HO and HA samples at day 1. **B** Volcano plot of 3 paired HO and HA samples at day 4. **C** Volcano plot of 3 paired HO and HA samples at day 7. **D** Heat map of 3 paired HO and HA samples at day 1. **E** Heat map of 3 paired HO and HA samples at day 4. **F** Heat map of 3 paired HO and HA samples at day 7. Different colors represent different expression levels (red high expression, white medium expression, and blue low expression). *DEGs* different expression genes, *HO* hematoma origination, *HA* hematoma absorbance

All the DEGs at days 1, 4 and 7 were showed in Additional files 1, 2 and 3. A total of 268 DEGs (117 up-regulated and 151 down-regulated) at day 1, 223 DEGs (91 up-regulated and 132 down-regulated) at day 4 and 17 DEGs (14 up-regulated and 3 down-regulated) at day 7 were found and illuminated by the volcano plots (Fig. 3A–C).

The heat maps using hierarchical clustering analysis showed that the expression patterns of mRNAs between the HA and HO samples were significantly different at day 1 (Fig. 3D), day 4 (Fig. 3E) and day 7 (Fig. 3F) postoperatively, which indicated that the tissue that the haematoma contacted with could determine the outcomes of the haematoma.

### GO and KEGG pathway analyses of DEGs

The details of the GO and KEGG analysis results were presented in Additional files 4 and 5.

**Fig. 4:**
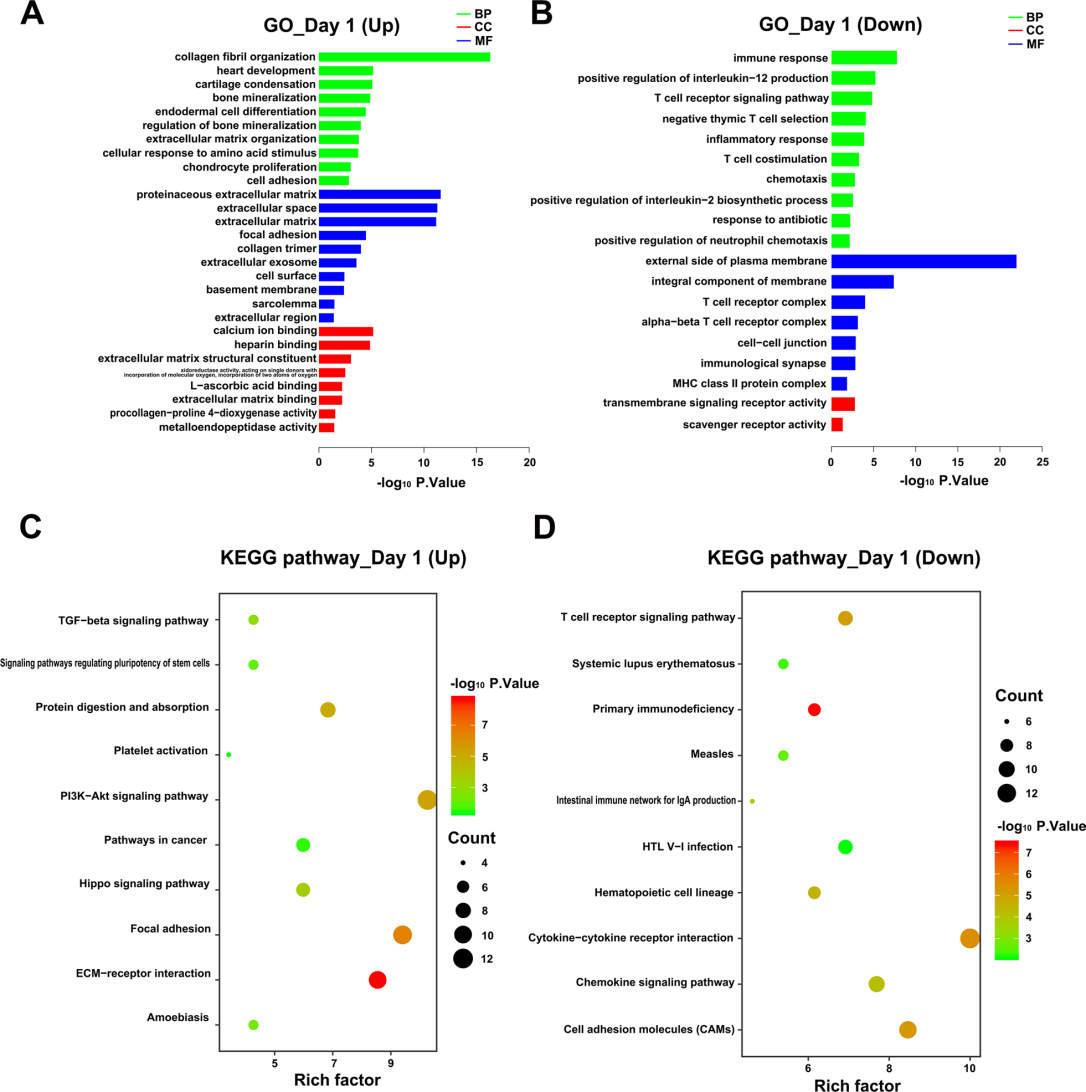
KEGG and GO enrichment analysis for day 1. **A**, **B**) Representative GO analysis of DEGs upregulated and downregulated DEGs respectively. The abscissa represents the − log10 (*p*.value) of enrichment term, and the ordinate represents the enrichment term’s names. **C**, **D** Representative KEGG pathway analysis of upregulated and downregulated DEGs respectively. The size of the circle represents gene count. The x-axis represents rich factor, and the y-axis represents pathway. Different circle colors represent different adjusted *p*.values. *GO* gene ontology, *BP* biological process, *CC* cellular component, *MF* molecular function, *KEGG* Kyoto encyclopedia of genes and genomes, *DEGs* different expression genes

At day 1, up-regulated DEGs could be enriched in 47 terms, including 28 BP terms, 11 CC terms and 8 MF terms (Additional file 4). In the BP domain, the most meaningful enriched GO terms primarily focused on collagen fibril organization (GO:0030199), cartilage condensation (GO:0001502), and bone mineralization (GO:0030282) (Fig. 4A). The most enriched CC terms were correlated to extracellular structure, such as extracellular space (GO:0005615), extracellular matrix (GO:0031012), and focal adhesion (GO:0005925) (Fig. 4A). For the MF terms, the represented terms were calcium ion binding (GO:0005509), heparin binding (GO:0008201), and extracellular matrix structural constituent activity (GO:0005201) (Fig. 4A). Furthermore, the KEGG pathway analysis indicated 10 KEGG pathways were significantly enriched and the up-regulated mRNAs were found to be associated with ECM-receptor interaction, Focal adhesion, PI3K-Akt signaling pathway and TGF-beta signaling pathway, Protein digestion and absorption (Fig. 4C).

Downregulated DEGs at day 1 could be enriched in 32 terms, including 23 BP terms, 7 CC terms and 2 MF terms (Additional file 4). Figure 4B displayed the most significant BP, CC, MF terms. Among them, the most representative GO-BPs terms of these DEGs were mainly involved in the immune and inflammatory responses, such as immune response (GO:0006955), positive regulation of interleukin-12 production (GO:0032735), T cell receptor signaling pathway (GO:0050852), negative thymic T cell selection (GO:0045060). At day 1, downregulated-DEGs significantly enriched in 17 pathways, and they were shown in bubble diagram (Fig. 4D), which were also almost related to immune response, such as Primary immunodeficiency, Cytokine-cytokine receptor interaction, Cell adhesion molecules, T cell receptor signaling pathway and Chemokine signaling pathway, etc.

**Fig. 5:**
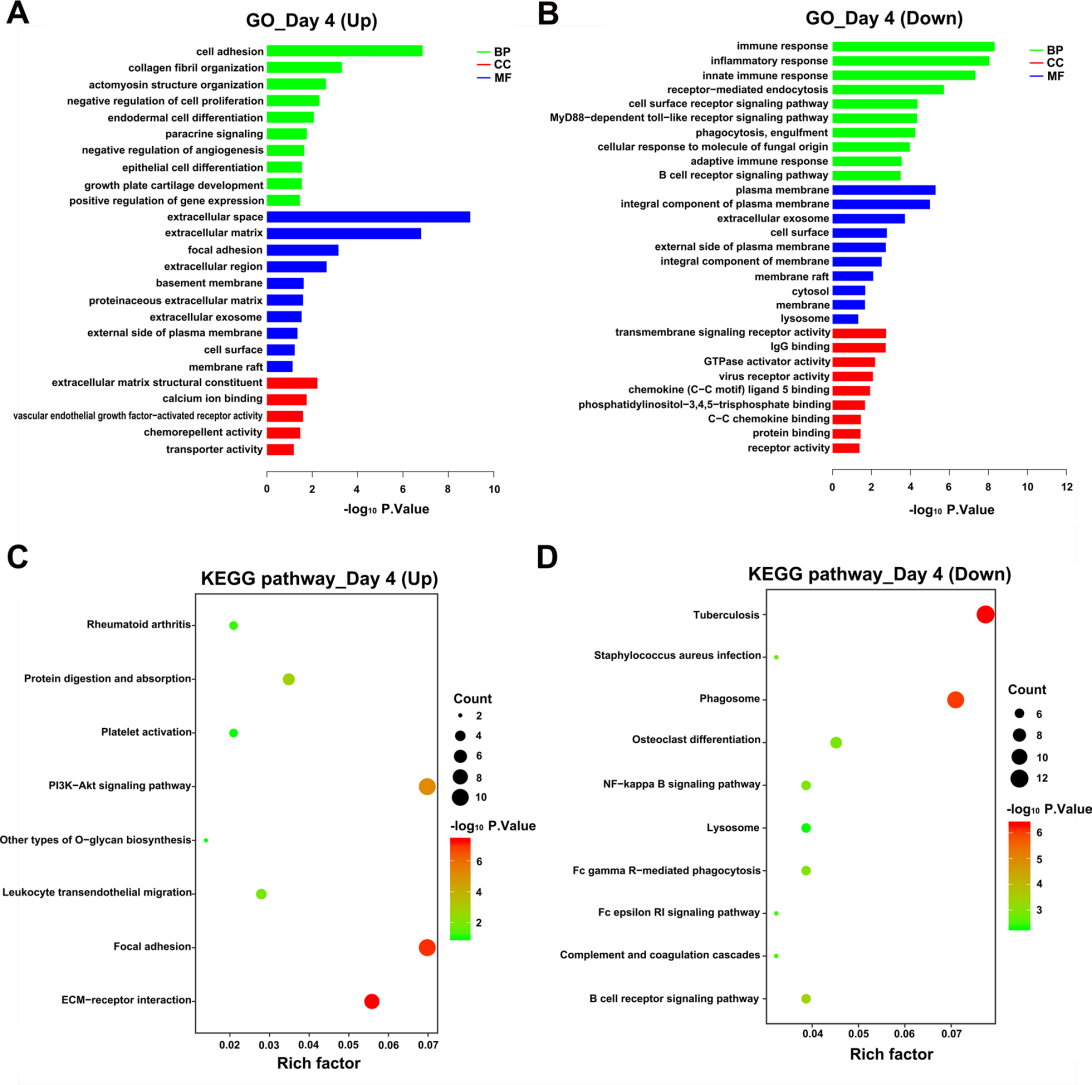
KEGG and GO enrichment analysis for day 4. **A**, **B** Representative GO analysis of DEGs upregulated and downregulated DEGs respectively. The abscissa represents the − log10 (*p*.value) of enrichment term, and the ordinate represents the enrichment term’s names. **C**, **D** Representative KEGG pathway analysis of upregulated and downregulated DEGs respectively. The size of the circle represents gene count. The x-axis represents rich factor, and the y-axis represents pathway. Different circle colors represent different adjusted *p*.values. *GO* gene ontology, *BP* biological process, *CC* cellular component, *MF* molecular function, *KEGG* Kyoto encyclopedia of genes and genomes, *DEGs* different expression genes

At day 4, the 91 upregulated DEGs could be grouped into 33 terms including 17 in BP, 11 in CC and 5 in MF through GO analysis (Additional file 5), and listed in the histogram (Fig. 5A). The BP terms were most relevant to cell adhesion (GO:0007155), collagen fibril organization (GO:0030199), actomyosin structure organization (GO:0031032), negative regulation of cell proliferation (GO:0008285) and growth plate cartilage development (GO:0003417). The CC terms was similar to the 1-day enrichment result, which was also mostly enriched in extracellular structure. The MF terms were closely related to extracellular matrix structural constituent (GO:0005201), calcium ion binding (GO:0005509). KEGG analysis of the 91 DEGs identified 9 pathways, they were shown in bubble diagram (Fig. 5C). Those pathways that enriched at day 1 (ECM-receptor interaction, Focal adhesion, PI3K-Akt signaling pathway and TGF-beta signaling pathway, Protein digestion and absorption) were also enriched at day 4.

At day 4, the 132 down-regulated DEGs could be grouped into 66 terms including 47 in BP, 10 in CC and 9 in MF through GO analysis (Additional file 5), and the representative terms were listed in the histogram (Fig. 5B). The BP terms were most relevant to immune response (GO:0006955) and inflammatory response (GO:0006954). Meanwhile a lot of terms related to phagocytosis were enriched, such as receptor-mediated endocytosis (GO:0006898), MyD88-dependent toll-like receptor signaling pathway (GO:0002755), phagocytosis, engulfment (GO:0006911), cellular response to molecule of fungal origin (GO:0071226). KEGG analysis identified 16 pathways (Additional file 5), and the representative 10 pathways were shown in bubble diagram (Fig. 5D). Several pathways were interesting, for example, Phagosome, B cell receptor signaling pathway, Fc gamma R-mediated phagocytosis and NF-kappa B signaling pathway.

At day 7, statistically significant GO term and KEGG pathway were not enriched due to the low number of DEGs.

### PPI network and module analysis

**Fig. 6:**
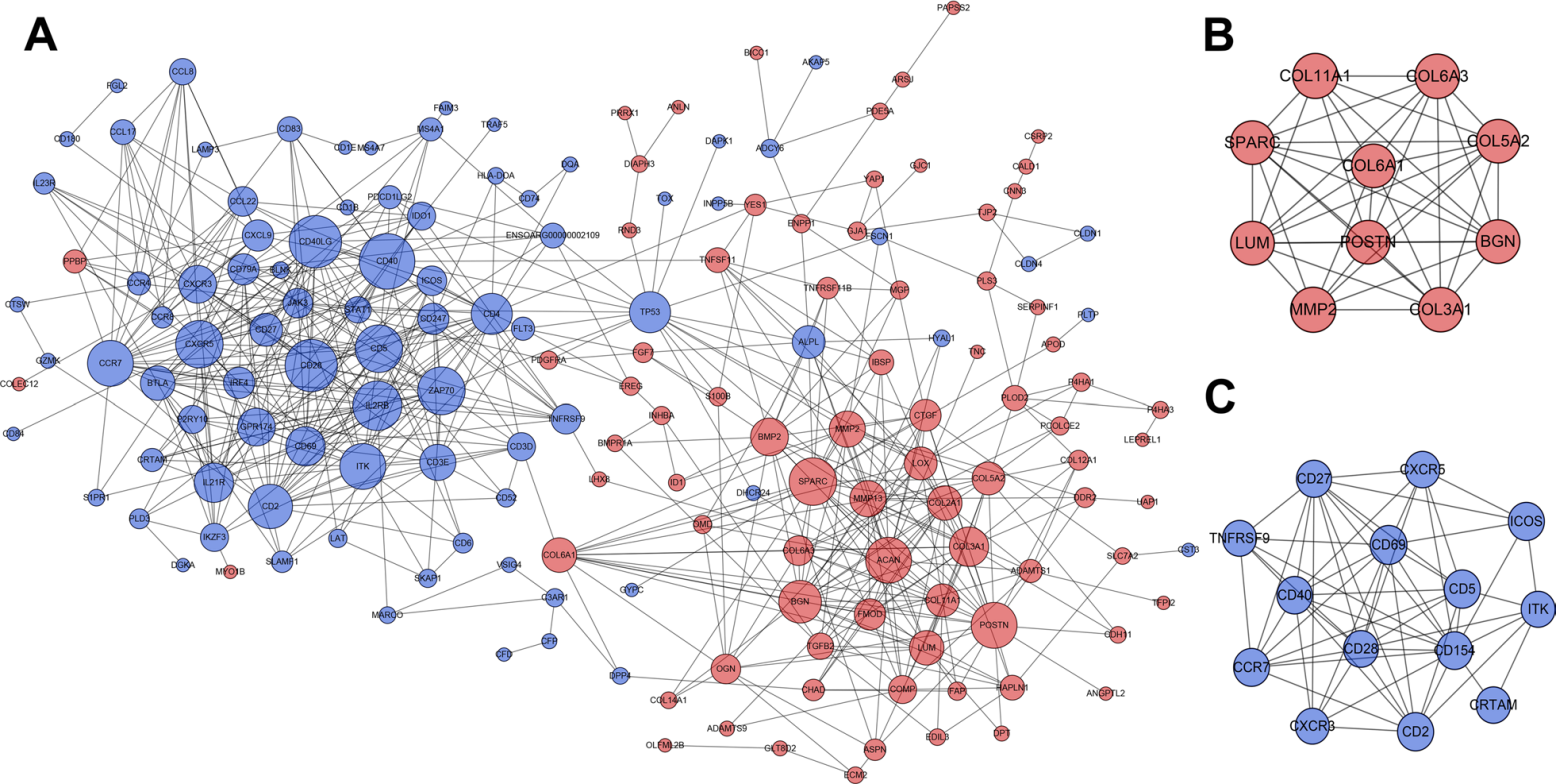
PPI network construction and Module analysis of the DEGs at day 1. **A** The PPI network of the DEGs consisted of 186 nodes and 614 edges. **B** The most significant module consisted of 10 nodes and 42 edges extracted from PPI network (MCODE score = 9.333). **C** The second significant module consisted of 14 nodes and 57 edges extracted from PPI network (MCODE score = 8.769). The nodes represent proteins, and the edges between nodes represent interactions. The greater the degree of correlation, the larger the diameter of nodes. The nodes are red for upregulated DEGs, and blue for downregulated DEGs. PPI, protein–protein interaction; DEGs, different expression genes

Based on the information of the STRING database, the PPI network of DEGs was constructed with 186 nodes and 614 edges (Fig. 6A) at day 1. By using MCODE analysis, 11 significant modules were screened out in the PPI network, and the most rewarding module (MCODE score = 9.333) consisted of 10 nodes and 42 edges (Fig. 6B). The module comprised POSTN, BGN, SPARC, LUM, MMP2 and some genes encoded collagen (COL3A1, COL5A2, COL6A1, COL6A3 and COL11A1), which were all upregulated DEGs. The second rewarding module (MCODE score = 8.769), consisting of 14 nodes and 57 edges (Fig. 6C), was composed of genes involved in immunity, such as CD40, CD28, CXCR5, TNFRSF9, etc. Interestingly, they were all downregulated DEGs.

**Fig. 7:**
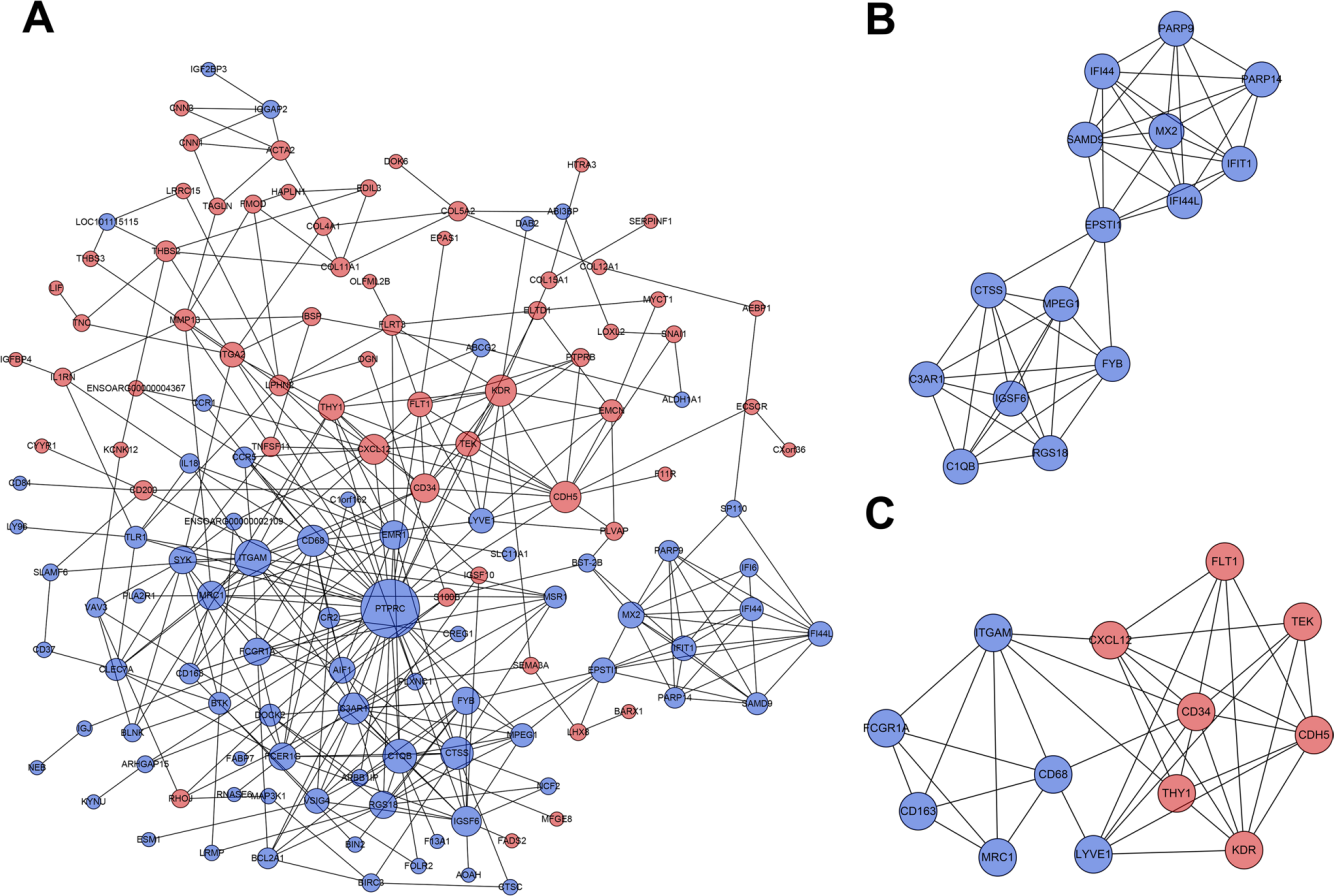
PPI network construction and Module analysis of the DEGs at day 4. **A** The PPI network of the DEGs consisted of 141 nodes and 356 edges. **B** The most significant module consisted of 15 nodes and 50 edges extracted from PPI network (MCODE score = 7.143). **C** The second significant module consisted of 13 nodes and 38 edges extracted from PPI network (MCODE score = 6.333). The nodes represent proteins, and the edges between nodes represent interactions. The greater the degree of correlation, the larger the diameter of nodes. The nodes are red for upregulated DEGs, and blue for downregulated DEGs. *PPI* protein–protein interaction, *DEGs* different expression genes

A total of 141 nodes and 356 edges were identified from the DEGs at day 4 (Fig. 7A). By using MCODE analysis, only 2 significant modules were screened out in the PPI network, and they were shown in Fig. 7B, C. The most rewarding module (MCODE score = 7.143) consisted of 15 nodes and 50 edges (Fig. 7B). The genes in this module were all down-regulated DEGs, and mainly associated with interferon (IFIT1, IFI44L, IFI44, and MX2) and macrophage (MPEG1). The second significant module was made up of both upregulated and downregulated DEGs (Fig. 7C). The upregulated DEGs in the module was mainly associated with angiogenesis, such as TEK, KDR, FIT1, and CXCL12. While the downregulated DEGs in the module were mainly related to the phagocytosis of macrophages, such as MRC1, CD163, CD68, and ITGAM.

### Verification of differentially expressed mRNAs by real time PCR

**Fig. 8:**
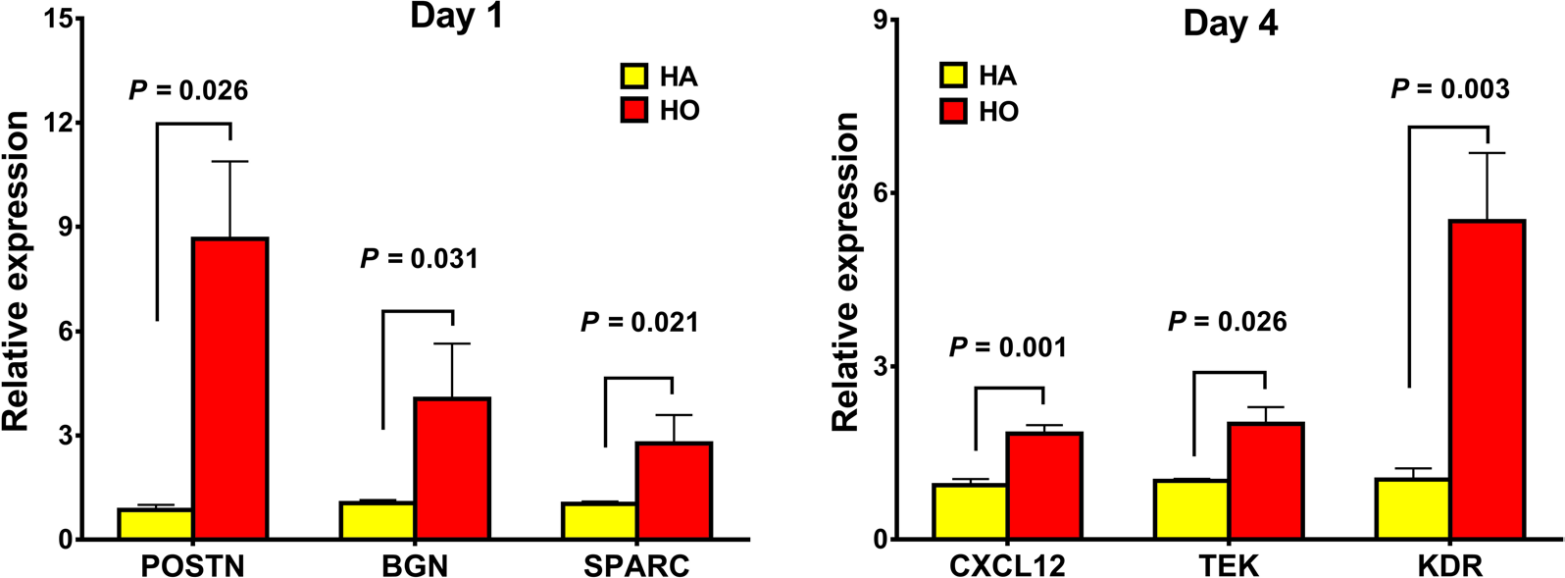
Real-time PCR analysis of the selected differentially expressed genes from the microarray results. Where given, the *p* values showed statistically significant differences between the hematoma origination side and hematoma absorbance side at the indicated time point

Six interesting DEGs were selected for confirmation by quantitative real-time PCR analysis. All of these 6 DEGs were genes in significant modules according to PPI analysis. We found that the expression pattern of the 6 genes (Fig. 8) was similar to the microarray results, demonstrating a good correlation between the two methods.

At day 1, POSTN (10.1-fold, *p* = 0.026), BGN (3.9-fold, *p* = 0.031), and SPARC (2.7-fold, *p* = 0.021) were up-regulated in HO in comparison to HA (Fig. 8).

At day 4, HO showed a significantly higher expression of CXCL12 (1.9-fold, *p* = 0.001), TEK (2.0-fold, *p* = 0.026), and KDR (5.3-fold, *p* = 0.003), compared to HA (Fig. 8).

## Discussion

Increasing evidences in TMJ ankylosis-related research have emphasized the importance of intra-articular haematoma, which possesses essential signals to initiate and activate healing processes [5, 23, 24]. In the present study, we explored the early pathological process of TMJ ankylosis in a sheep model by removal of disk and articular fibrous layers. The intra-articular haematoma organized into granulation tissue in the joint space at days 4 and 7 postoperatively. As a control, the blood clot in the joint space did not organize and was almost absorbed in the HA side at day 7 after surgery, which was in accordance with the arthroscopy of acute TMJ trauma of human beings [6].

The fate of experimental haemarthrosis, whether absorbance or organization into ankylosis, depended on the tissue it contacted in the sheep model. Namely, if haemarthrosis keeps in touch with the fibrous layers, it will be absorbed. While if haemarthrosis contacts and interactions with the proliferative zone cells or chondrocytes, ankylosis develops. The proliferation zone cells and articular chondrocytes might secrete cytokines and growth factors into the haematoma. Or the osteoprogenitor cells underneath the fibrous layers might migrate into the joint space mediated by the haematoma. These factors will promote the development of TMJ ankylosis. However, in the HA side, due to the barrier function of the fibrous layer, no foreign cells and growth factors can reach to and interact with the haematoma, therefore presenting with a natural course of haematoma absorbance. To explain the different outcomes of haematoma in the animal model, a genome-wide transcriptional analysis of haemarthrosis was performed, and a series of significant genes, pathways and molecular events during the onset and progress of TMJ ankylosis were identified.

A total of 268 and 223 DEGs between the HO and HA samples were screened at days 1 and 4 respectively. The large amount of DEGs at days 1 and 4 reflected the obviously differences between the haematoma absorbance and organization in the joint space. However only 17 DEGs were found at day 7. It seemed that there should also be a large number of DEGs at day 7 since the haemarthrosis was subsequently absorbed completely at days 9 and 11 in the disk removal side, while the hematoma was highly organized in the ankylosis-induced side at the same time point. The reason, we speculated, might be that the outcome of blood clot in the disk removal side was not simply absorbed in the joint space, but partly participated in the repair of the joint capsule. While the hematoma in the ankylosis-induced side not only repair of the joint capsule, but also organized in the joint space, therefore ultimately leading to TMJ ankylosis. Therefore the relatively less DEGs at day 7 indicated the similarity of repair of joint capsule between the 2 sides. The pathological results at day 7 confirmed our speculation.

At day 1, as revealed by GO and KEGG pathway analysis, up-regulated DEGs in the ankylosis-induced side were enriched in the biological processes of collagen fibril organization and new bone formation, while at day 4 most overrepresented GO-BPs terms of up-regulated DEGs were mainly involved in cell adhesion, collagen fibril organization, and growth plate cartilage development. According to our previous study, the development of the TMJ ankylosis is similar to the mal-union of fracture [25]. In the present study, a large blood clot filled the joint space immediately after trauma due to the removal of the disc and the fibrous layers of the condylar head and glenoid fossa, as well as the tear of the attachment around the joint. Thrombase produced by blood vessel rupture induced fibrinogen in the blood to break down subsequently, forming fibrinogen monomers which are then rapidly aggregated. In this way, the earliest fibrin net, will be the first bridge among the traumatic articular surfaces within 6–8 h [26, 27]. As soon as a fibrin clot begins to form, mesenchymal stem cells (MSCs) will be recruited and give rise to fibroblasts. Once the fibroblasts are attached to the fibrin net, they secrete a large amount of collagen, including COL3A1, COL5A2, COL6A1, COL6A3 and COL11A1 as shown in the present study, between 24 and 48 h after trauma [28]. In this collagen matrix, endothelial cells proliferate and gradually form new networks of capillaries. At the same time, TGF-β pathway, which was produced by granulation tissue to regulate the proliferation of fibroblasts and collagen synthesis [29], was also highly expressed in the HO side. In addition, BMP-2, as one member of TGF-β pathway, can also induce the further differentiation of stem cells into osteoblasts [30]. Meanwhile, apoptosis was inhibited following PI3-Akt pathway activation [31, 32]. Under the multiple effects of the above factors, the new tissue generated in the joint cavity.

Regarding the results in the HA side, we found a large number of neutrophils gathered at the surgical site within the first 24 h after surgery. BP terms such as immune response, T cell receptor signaling pathway, T cell costimulation and chemotaxis were enriched. Both histoligical and GO analysis showed that the HA side had a more severe inflammatory response than the HO side. Similarly, fibrin scaffolds and collagen matrix also formed in the joint space in the HA side. However, it seemed that they only served as scaffolding for the infiltrating cells (leukocytes, keratinocytes and fibroblasts) [33], since the intact articular surfaces prevented the key signaling factors regulating collagen mineralization and osteogenic differentiation of MSCs from entering the haematoma. More importantly, a large number of macrophages migrated to the site of injury and began to remove debris, necrotic tissue and pathogens at the site of injury during 48–96 h after surgery [34]. At this point, several interesting genes had higher expression in the HA side, for example, CD86 (promoting phagocytosis and removal of cell debris by macrophages [35]), MRC1 (promoting endosytosis of glycosylated protein and collagen in macrophages [36, 37]), and CD163 (participating in phagocytosis and phagocytosis of hemoglobin/haptoglobin complex [38]). This also explained why the haematoma was absorbed from the molecular level.

In the present study, up-regulated DEGs in significant modules screened by PPI, such as POSTN, BGN, SPARC, and LUM at day 1, and TEK, KDR, FLT1 at day 4, played an important role in this process above-mentioned.

At day 1, we found Periostin (POSTN) significantly up-regulated in the HA samples. Firstly identified in murine osteoblasts, this gene is now discovered to be specifically expressed in collagen-rich tissues, such as periodontal ligament and periosteum, and to transfer mechanical stress [39]. POSTN acts as a structural component of the matrix regulating collagen cross-linking and as a signaling molecule via interaction with integrin receptors and Wnt/β-catenin pathways to promote osteoblast functions [40]. POSTN is considered a key extracellular matrix protein needed in healing. It also plays an important role in promoting periosteal callus formation and repair of bone fractures during the early stage of fracture healing [41]. In the present study, the up-regulated expression of POSTN in the HA at day 1 postoperatively indicated an important role in the onset of TMJ ankylosis, and deserved to be further studied.

Biglycan (BGN), Fibromodulin (FMOD) and lumican (LUM) are subtypes of the small leucine-rich family of proteoglycans (SLRP) [42]. SLRPs are the major non-collagen components of the extracellular matrix, with the function of affecting bone growth, craniofacial structure, dentin formation and collagen production [43]. Both BGN and FMOD are critical for the differentiation and function of TMJ chondrocytes by modulating TGF-β1 activity [44]. BGN deletion may cause structural abnormalities of bone collagen fibrils and impair the metabolic activity of bone marrow stromal cells (BMSCs) [45, 46]. Considering a role in early collagen fibril assembly [47], LUM might be involved in both inflammation and remodelling in response to dermal injury [48].

SPARC is a 32 kDa calcium-binding matricellular protein, also referred to as osteonectin or basement membrane protein 40 (BM-40) [49]. During bone formation, SPARC is secreted by osteoblasts [50]. In osteoid, SPARC is thought to bind collagen and hydroxyapatite crystals and release calcium ions that promote the mineralization of the collagen matrix [51]. Notably, SPARC is expressed at high levels in bone remodeling process than steady bone tissue [52]. Delany et al. [53] have reported that osteoblastic precursors were reduced in SPARC-null mice, and that the capacity of osteoblast differentiation of bone marrow derived cells from SPARC-null was impaired.

All of the above molecules are expressed in the extracellular matrix and can participate in the reconstruction and assembly of collagen and affect the next step of bone mineralization, suggesting that extracellular matrix plays an important role in the occurrence of TMJ ankylosis. The presumption is in accordance with the results of CC in our GO analysis.

At day 4, the genes regulating angiogenesis (TEK, KDR, FLT1) were screened by PPI analysis. The process of angiogenesis is mainly regulated by two molecular pathways. One is angiopoietin-dependent pathway, the other is vascular endothelial growth factor (VEGF)-dependent pathway [54]. Their expression was induced early in the healing cascade, suggesting that they promoted initial vascular growth of existing vessels in the periosteum [54]. TEK (also known as Tie2) is the tyrosine kinase receptor of Angiopoietin-1 (Ang 1) and Angiopoietin-2 (Ang 2) in the angiotensin signaling pathway [55]. VEGF binds to tyrosine kinase cell receptors, including VEGFR-1 (Flt-1), and VEGFR-2 (KDR). VEGFR-1 and VEGFR-2 are expressed predominantly on vascular endothelial cells, with strong pro-angiogenic activity [56]. These results suggest that angiogenesis was significantly enhanced at day 4 in the HO side, which is also consistent with our histological results.

Among the up-regulated genes screened by PPI analysis in the ankylosis-induced side at day 4, a key gene, CXCL12, is worth noting. It is a key inflammatory cytokine and may be involved in the initial inflammatory response as the first step of fracture healing. As an important chemokine, CXCL12 has been identified to regulate the inflammatory response associated with the healing process [57]. Furthermore, it is an important contributor to bone marrow MSC homing and localization within the bone marrow [58]. Our findings indicated that the early expression of CXCL12 could promote the genesis of TMJ ankylosis, therefore might be an promising target for the prevent of TMJ ankylosis in the future.

This study has some limitations. First, the number of experimental animals is too small, which may lead to an increase in type 2 errors in statistics. Secondly, the whole-body circulation of cytokines/inflammation response might affect bilateral injury sites since the ips- and contra-lateral tissue samples were obtained from the same animal. However, the animal model was reliable, and the obvious histological differences between the HA and HO side was confirmed in the present study. Therefore, the molecular basis underlying the histological differences, as uncovered by microarray analysis of DEGs, was convincing. Finally, the key genes and signaling pathways obtained based on bioinformatics methods are only a preliminary study, and further basic experiments and clinical validation are needed in the future.

In conclusion, our study demonstrated the DEGs between the HO and HA samples at several time points postoperatively by using an extensive bioinformatics analysis. Genes that promoted collagen ossification (POSTN, BGN, LUM, SPARC) and angiogenesis (KDR, FIT1, TEK) had a positive effect on the onset and progress of TMJ ankylosis. Further investigation of the precise functional roles of these genes will provide new ideas for future treatment and prevention of the disease.

## Supplementary Information

**Additional file 1.** DEGS at day 1.

**Additional file 2.** DEGS at day 4.

**Additional file 3.** DEGS at day 7.

**Additional file 4.** GO and KEGG pathway analyses of DEGS at day 1.

**Additional file 5.** GO and KEGG pathway analyses of DEGS at day 4.

## Data Availability

The datasets used and/or analyzed during the current study are available from the corresponding author on reasonable request.

## Abbreviations

TMJ: Temporomandibular joint
HA: Haematoma absorbance
HO: Haematoma organization
DEGs: Different expression genes
PPI: Protein–protein interaction
FC: Fold-change
GO: Gene ontology
BP: Biological process
MF: Molecular function
CC: Cellular component
DAVID: Database for annotation visualization and integrated discovery
KEGG: Kyoto encyclopedia of genes and genomes
STRING: Search tool for the retrieval of interacting genes/proteins
MCODE: Molecular complex detection
PCR: Reverse transcription and real-time polymerase chain reaction
GAPDH: Glyceraldehyde-3-phosphate dehydrogenase
POSTN: Periostin
BGN: Biglycan
SPARC: Secreted protein acidic and cysteine rich
LUM: Lumican
MMP2: Matrix metallopeptidase 2
COL3A1: Collagen type III alpha 1 chain
COL5A2: Collagen type V alpha 2 chain
COL6A1: Collagen type VI alpha 1 chain
COL6A3: Collagen type VI alpha 3 chain
COL11A1: Collagen type XI alpha 1 chain
CD40: CD40 molecule
CD28: CD28 antigen
CXCR5: C-X-C motif chemokine receptor 5
TNFRSF9: TNF receptor superfamily member 9
IFIT1: Interferon induced protein with tetratricopeptide repeats 1
IFI44L: Interferon induced protein 44 like
IFI44: Interferon induced protein 44
MX2: MX dynamin like GTPase 2
MPEG1: Macrophage expressed 1
TEK: TEK receptor tyrosine kinase
KDR: Kinase insert domain receptor
FIT1: FMS related receptor tyrosine kinase 1
CXCL12: C-X-C motif chemokine ligand 12
MRC1: Mannose receptor C-type 1
CD163: CD163 molecule
CD68: CD68 molecule
ITGAM: Integrin subunit alpha M
MSCs: Mesenchymal stem cells
BMP-2: Bone morphogenetic protein 2
TGF-β: Transforming growth factor, beta
FOMD: Fibromodulin
SLRP: Small leucine-rich family of proteoglycans
VEGF: Vascular endothelial growth factor
Ang 1: Angiopoietin-1
Ang 2: Angiopoietin-2
